# Decreased Krüppel-like factor 4 in adenomyosis impairs decidualization by repressing autophagy in human endometrial stromal cells

**DOI:** 10.1186/s12860-022-00425-6

**Published:** 2022-06-27

**Authors:** Jie Mei, Xiaoqiang Sheng, Yuan Yan, Xinyu Cai, Chunxue Zhang, Jiao Tian, Mei Zhang, Jidong Zhou, Huizhi Shan, Chenyang Huang

**Affiliations:** 1 Center for Reproductive Medicine and Obstetrics and Gynecology, Nanjing Drum Tower Hospital, Nanjing University Medical School, Nanjing, 210008 China; 2 Center for Molecular Reproductive Medicine, Nanjing University, Nanjing, 210008 China; 3 Department of Obstetrics and Gynecology, Nanjing First Hospital, Nanjing Medical University, Nanjing, 210006 China

**Keywords:** Krüppel-like factor 4, Adenomyosis, Impaired decidualization, Autophagy, Transcriptional regulation, Autophagy-related 5

## Abstract

**Background:**

Poor decidualization and abnormal autophagy conditions in the endometria of adenomyosis patients have been reported previously. However, the specific regulatory mechanism of decidualization in adenomyosis and its relationship with autophagy levels have not been clarified.

**Methods:**

Endometrial tissues from adenomyosis patients and uteri from an adenomyosis mouse model were collected for the detection of different expression patterns of KLF4 and autophagy markers (LC3-B/LC3-A and Beclin-1) compared with control groups. Human endometrial stromal cells (hESCs) isolated from adenomyosis and control endometrial tissues were employed to elucidate the biological functions of KLF4 in autophagy and decidualization. Gene expression regulation was examined by quantitative real-time PCR (qRT-PCR), western blotting and luciferase reporter assays. In addition, DNA promoter-protein interactions were examined by chromatin immunoprecipitation (ChIP)/PCR assay and avidin–biotin conjugate DNA precipitation (ABCD) assay.

**Results:**

KLF4 expression was decreased in endometrial tissues from adenomyosis patients compared with those from fertile controls, especially in stromal compartments. The opposite results were observed for autophagy marker (LC3-B/LC3-A and Beclin-1) expression. At the same time, KLF4 reversed the poor decidualization of hESCs from adenomyosis patients. In addition, KLF4 could induce hESC decidualization by promoting the autophagy level. Mechanistically, KLF4 bound to a conserved site in the autophagy-related 5 (ATG5) promoter region and promoted ATG5 expression. Similar expression patterns of KLF4 and autophagy markers were detected in adenomyotic mice.

**Conclusions:**

KLF4 overexpression increases the autophagy level of hESCs by transcriptionally promoting ATG5 expression, and abnormally decreased KLF4 in adenomyosis impairs hESC decidualization by repressing autophagy.

**Supplementary Information:**

The online version contains supplementary material available at 10.1186/s12860-022-00425-6.

## Background

Adenomyosis can affect clinical pregnancy, and is one of the critical factors leading to infertility [1]. It is also considered an important reason for the decrease in pregnancy rate and the increase in miscarriage rate in assisted reproductive technology (ART) cycles [2, 3]. The incidence rate of adenomyosis is increasing gradually and tends to occur in younger women. The incidence rate of adenomyosis is approximately 15%, which can reach as high as 54% of patients who turn to ART treatment. Studies have shown that adenomyosis has adverse impacts on embryo implantation, but the exact mechanism is currently unclear [4].

Successful embryo implantation depends on the appropriate interaction between high-quality embryos and receptive endometrium [5]. Under the control of ovarian oestrogen and progesterone, endometrial epithelial cells enter the "receptive state" to initiate the first maternity-foetus dialogue; at the same time, the process of endometrial stromal cell decidualization initiates, which is identified as a prerequisite for further embryo invasion and development [5–7]. Studies have found that the endometrial receptivity of adenomyosis patients is impaired, which is mainly manifested by the abnormal expression of embryo adhesion related factors ( homeobox A10 (HOXA10), leukaemia inhibitory factor (LIF), and cytochrome P450) [8–10] and the inhibition of endometrial stromal cell decidualization [11]. Therefore, abnormal decidualization may be one of the important factors affecting endometrial receptivity in adenomyosis, and the regulatory mechanism needs further study. Decidualization is a process in which endometrial stromal cells transform from initial fibroblast-like cells to specific decidual secretory cells with the remodelling of the stromal cytoskeleton [12, 13]. Decidualized stromal cells can provide a nutrient-supplied and immunotolerant microenvironment for successful embryo implantation and formation of the placenta [14, 15].

Several members of the Krüppel-like factor family expressed in the endometrium have been confirmed to be regulated by oestrogen/progesterone and participate in embryo implantation (KLF5 and KLF9) [16–19]. In addition, our previous study suggested that KLF12 could affect the decidualization process by transcriptional inhibition of the expression of the decidualization-related factors NR4A1 and FOXO1A, which leads to embryo implantation failure [20, 21]. In our further study, we found that KLF4, another member of the KLF family, could also regulate the decidualization of endometrial stromal cells. At the same time, we detected abnormally decreased expression of KLF4 protein in the endometrium of infertile patients with adenomyosis. Recent studies have shown that the autophagy condition of endometrial cells is closely related to embryo implantation and the maintenance of pregnancy. The expression of the autophagy marker protein LC3-B in the decidua and trophoblasts of normal pregnancy was significantly higher than that of patients experiencing embryo implantation failure or early miscarriage [22]. The process of artificially induced decidualization of the uterus with low autophagy was inhibited in an obesity mouse model [23]. Therefore, normal autophagy conditions may be crucial for embryo implantation and decidualization of endometrial stromal cells with low autophagy conditions may be repressed, resulting in abnormal pregnancy. Abnormal changes in autophagy conditions in the endometria of adenomyosis patients have been reported. The endometria of patients with adenomyosis are under conditions of high oestrogen stimulation and progesterone resistance, which represses the local autophagy condition through the mammalian target of rapamycin (mTOR) pathway [24]. Contrary to the expression pattern of KLF4, we found that the autophagy levels of endometrial stromal cells were significantly lower in infertile women with adenomyosis (decreased expression of LC3-B/LC3-A and Beclin-1) than in fertile women, similar to other studies [25, 26]. Therefore, we hypothesized that abnormally decreased KLF4 expression in patients with adenomyosis leads to low autophagy levels in endometrial stromal cells, which is one of the important reasons for abnormal decidualization.

In this study, we identified KLF4 as a key decidualization regulator, that promotes the decidualization process of endometrial stromal cells by transcriptionally inducing expression of the ATG5 protein. In addition, we found that the level of KLF4 in the eutopic endometrium of patients with adenomyosis was abnormally decreased and that the autophagy level was abnormally repressed. Therefore, these results revealed that abnormally decreased KLF4 expression triggers low autophagy levels of the endometria of patients with adenomyosis, which impairs endometrial decidualization and leads to embryo implantation failure.

## Methods

### Patient sample collection

Endometrial samples were collected from patients who received in vitro fertilization (IVF) treatment in the reproductive medicine centre of Nanjing Drum Tower Hospital. There were 12 infertile patients with adenomyosis and 12 patients with successful pregnancy in the control group. The detailed information of these patients is listed in Table 1, and there were no significant differences. None of the patients received oral contraceptives 3 months before the operation. Endometrial tissues were obtained by endometrial biopsy 5–7 days after ovulation. The diagnostic criteria for adenomyosis were described in our previous studies [27]. The exclusion criteria were as follows: polycystic ovary syndrome (PCOS), untreated hydrosalpinx, endometrial polyps and endometrial lesions.

**Table 1:** Demographic details of the participants in this study

Disease	Normal (n=12)	Adenomyosis (*n*=12)	*P* value
Age (years)	30.1 ± 0.8	30.5 ± 0.7	NS
Body mass index (kg/m^2^)	22.7 ± 0.5	22.9 ± 0.7	NS
Menstrual cycle (days)	28.6 ± 0.3	29.4 ± 0.4	NS

### Isolation and decidualization stimulation of human endometrial stromal cells (hESCs)

As mentioned in our previous study [11, 20], primary human endometrial stromal cells were isolated from the endometria of infertile patients with adenomyosis and control group patients. hESCs were cultured according to the routine [11, 20]. To induce decidualization, the hESCs were cultured in phenol red-free DMEM/F12 medium (HyClone, Thermo Scientific, South Logan, UT, USA) containing 2.5% (v/v) charcoal/dextran-treated foetal bovine serum (FBS; HyClone, Thermo Scientific, South Logan, UT, USA), 100 IU/ml penicillin, and 100 μg/ml streptomycin supplemented with 0.5 mM 8-Br-cAMP (Sigma, St. Louis, MO, USA) and 1 μM medroxyprogesterone acetate (MPA, Sigma, St. Louis, MO, USA) to induce decidualization in vitro. After stimulation for different times, the culture supernatant was collected to detect decidual prolactin (dPRL) levels and evaluate the degree of decidualization. To overexpress KLF4 or reduce KLF4 expression, hESCs were treated with Ad-His-KLF4 and SiKLF4 for 1–2 days before decidualization stimulation. To explore the effect of autophagy inhibitors on decidualization, cells were pretreated with 3-methyladenine (3-MA, MedChemExpress, Shanghai, China) 2 days before decidualization stimulation.

### Oligonucleotide transfection

The siRNA duplexes targeting human KLF4 (SiKLF4: GAGAGACCGAGGAGTTCAA) and siRNA negative control oligonucleotides were synthesized by RiboBio (Guangzhou, China). The siRNA negative control shared a homologous region with the human genome sequences. Oligonucleotide transfection was performed in hESCs using Lipofectamine 3000 (Life Technologies, New York, USA) according to the manufacturer’s instructions. At 48 h posttransfection, the cells were collected or decidualization was induced for the indicated time [27].

### RNA isolation and quantitative real-time PCR

Human endometrial stromal cells were lysed with TRIzol reagent (Sigma, St. Louis, MO, USA) and total RNA was extracted in strict accordance with the instructions. Two micrograms of total RNA was added to a 20 μL system and reverse-transcribed into cDNA using 5 × All-In-One RT Master Mix (Abm, Canada). A SYBR Green PCR kit and MyiQ Single Colour Real-time PCR Detection System (Bio-Rad Laboratories, Hercules, CA, USA) were employed for quantitative real-time PCR (qRT-PCR). The primer sequences used were as follows: KLF4, 5′-AGAGTTCCCATCTCAAGGCA-3′ and 5′-GTCAGTTCATCTGAGCGGG-3′; ATG5, 5′-AAAGATGTGCTTCGAGATGTGT-3′ and 5′-CACTTTGTCAGTTACCAACGTCA-3′; dPRL, 5′-CACTACATCCATAACCTCTC-3′ and 5′-ATGCTGACTATCAAGCTCAG-3′; IGFBP-1, 5′-TATGATGGCTCGAAGGCTCTC-3′ and 5′-GTAGACGCACCAGCAGAGTC-3′ and 18S rRNA, 5′-CGGCTACCACATCCAAGGAA-3′ and 5′-CTGGAATTACCGCGGCT-3′. The fold changes in the expression of each gene were measured by the 2^−ΔΔCT^ method. The internal reference gene was 18S rRNA.

### Western blot

According to our previous studies [20], we extracted and measured the total protein of endometrial tissue, hESCs and uteri of mice with or without adenomyosis. The protein concentrations were measured by the Bradford assay (Bio-Rad, Hercules, CA, USA). A fixed amount of protein (25–40 μg) was used for SDS polyacrylamide gel (10%-15%) separation electrophoresis and then transferred to polyvinylidene fluoride (PVDF) membrane. Immunoblotting was carried out by incubating with the primary antibodies and horseradish peroxidase (HRP)-conjugated secondary antibodies. The specific antibody information was as follows: anti-KLF4 (1:1000; 11,880–1-AP, rabbit polyclonal antibody, Proteintech Group, USA), anti-Beclin-1 (1:1000; 11,306–1-AP, rabbit polyclonal antibody, Proteintech Group, USA), anti-LC3A (1:1000; 18,722–1-AP, rabbit polyclonal antibody, Proteintech Group, USA), anti-LC3B (1:1000; 18,725–1-AP, rabbit polyclonal antibody, Proteintech Group, USA), anti-ATG5 (1:1000; 10,181–2-AP, rabbit polyclonal antibody, Proteintech Group, USA), anti-GAPDH (1:10,000; AP0063, GAPDH polyclonal antibody, Bioworld Technology, MN, USA) and goat anti-rabbit horseradish peroxidase (HRP)-conjugated secondary antibody (1:10,000; BS13278, Bioworld Technology, St. Louis Park, MN, USA). An enhanced chemiluminescence kit (Amersham Biosciences Corp., Piscataway, NJ, USA) was used for detection and Quantity-one (Bio-Rad Laboratories, Hercules, CA, USA) software was used for density analysis of each band.

### Immunofluorescence staining for F-actin filaments

According to our previous research [20], hESCs grown in 8-well chambers (Millipore, Billerica, MA, USA) were treated with adenovirus, siRNA or autophagy inhibitor for 2 days, and decidualization was induced by 8-Br-cAMP + MPA for 3 days. Then, according to our previous research [20], hESCs were fixed with 4% paraformaldehyde (w/v) for 30 min at room temperature. Next, the cells were washed with PBS and permeabilized with 0.5% Triton X-100 in PBS at room temperature. Subsequently, the cells were blocked with 3% BSA in PBS and incubated with fluorescein isothiocyanate-labelled phalloidin (1:300; P5282, Sigma, St. Louis, MO, USA) at 4 °C overnight. Cell nuclei were stained with DAPI (5 μg/ml) on the following day. The final images were taken with a confocal laser microscope (Leica, Wetzlar, Germany).

### Luciferase assays

The sequence (-2932 bp to + 100 bp) containing the KLF4 specific binding site in the ATG5 promoter region was inserted into the pGL3-basic luciferase reporter plasmids. At the same time, the sequence (-1859 bp to + 100 bp) without the KLF4-binding site was inserted into the pGL3-basic luciferase reporter plasmid. Human endometrial stromal cells cultured in a 12-well plate were infected with Ad-His-KLF4 and then transfected with 300 ng luciferase reporter plasmid by Lipofectamine 3000 (Life Technologies, Carlsbad, CA, USA). After 48 h, a dual luciferase analysis system (Promega, Madison, WI, USA) was used to analyse the luciferase activity. A luminescent counter (Centro xs3 LB 960, Berthold Technologies) was used to measure the luciferase activity.

### Chromatin immunoprecipitation (ChIP)/PCR assay

hESCs (70% confluence) were infected with Ad-LacZ or Ad-His-KLF4 (at a multiplicity of infection (MOI) of 50) for 48 h and then maintained in phenol red-free DMEM/F12 medium containing 2.5% charcoal/dextran-treated FBS with 0.5 mM 8-Br-cAMP plus 1 μM MPA. After 72 h, the hESCs were prepared for ChIP as described previously [20, 28]. Crosslinking, cell lysis, and genomic DNA fragment extraction were performed and KLF4 antibody was used for immunoprecipitation.The recovered DNA was analysed by RT-PCR. Specific primers (ATG5 5’-ATGGCCATCGTGAACACGTC-3’ and 5’-CAAATCAGTGGCACTGCAAA-3’) containing the KLF4-binding sequence were used for PCR amplification of ATG5 promoter fragments and a negative control primer (targeting: − 8000 bp to − 7790 bp) was also employed.

### Avidin–biotin conjugate DNA precipitation (ABCD) assay

Double-stranded oligonucleotides were designed based on the ATG5 promoter sequence (− 2644 to − 2605 bp). The 5′ end of the sense strand was biotinylated, and a deletion and a mutation were introduced (deletion and mutation of the CACCC sequence) to remove the specific binding site for KLF4. The following primers were designed: human ATG5 wild type: 5′-biotin-GTTCCCAACAGAGAGTCACCCCCAATAAGCTAAAACTTGG-3′; human ATG5 wild-type reverse: 5′-CCAAGTTTTAGCTTATTGGGGGTGACTCTCTGTTGGGAAC-3′; human ATG5 del: 5′-biotin-GTTCCCAACAGAGAGTCCAATAAGCTAAAACTTGG-3′; human ATG5 del reverse: 5′-CCAAGTTTTAGCTTATTGGACTCTCTGTTGGGAAC-3′; human ATG5 mut: 5′-biotin-GTTCCCAACAGAGAGTCTGACCCAATAAGCTAAAACTTGG-3′; human ATG5 mut reverse: 5′-CCAAGTTTTAGCTTATTGGGTCAGACTCTCTGTTGGGAAC-3′. hESCs were infected with Ad-LacZ and Ad-His-KLF4 (50 MOI) for 48 h. The ABCD method was performed as described in our previous research [20, 28]. Cell extracts were harvested and lysed in RIPA buffer. Each double-stranded DNA sample (500 pmol) was incubated with 500 μg of cell extract at 4 °C for 2–4 h, and the protein complexes were pulled down using streptavidin agarose beads (Sigma) in binding buffer (10 mM Tris, pH 8.0; 150 mM NaCl; 0.5% Triton X-100; 0.5 mM DTT; 0.5 mM EDTA; 10% glycerol; and 20 μg/mL poly [dI–dC]) containing a protease inhibitor cocktail. The proteins were eluted, separated by SDS–PAGE, and then probed with KLF4 antibody (1:1000; 11,880–1-AP, rabbit polyclonal antibody, Proteintech Group, USA) and the corresponding secondary antibody.

### Immunohistochemistry

Fresh endometrial tissue and mouse uterine tissue were fixed, embedded in paraffin and serially sectioned (5 μm). Formalin-fixed, paraffin-embedded uterine endometria were serially sectioned, dewaxed with xylene and rehydrated through a graded alcohol series, and then endogenous peroxidase activity was blocked using freshly prepared phosphate-buffered saline (PBS) containing 3% hydrogen peroxide for 20 min. Antigen retrieval was conducted by autoclaving the samples at 121 °C for 15 min in the presence of EDTA (pH 9.0), followed by incubation in blocking solution for 30 min. Next, the sections were washed with PBS and incubated with the specific primary antibodies overnight at 4 ℃ [20]. The specific antibody information was as follows: anti-KLF4 (1:500; 11,880–1-AP, rabbit polyclonal antibody, Proteintech Group, USA), anti-Beclin-1 (1:500; 11,306–1-AP, rabbit polyclonal antibody, Proteintech Group, USA), anti-LC3A (1:500; 18,772–1-AP, rabbit polyclonal antibody, Proteintech Group, USA), anti-LC3B (1:500; 18,725–1-AP, rabbit polyclonal antibody, Proteintech Group, USA), anti-ATG5 (1:500; 10,181–2-AP, rabbit polyclonal antibody, Proteintech Group, USA). Subsequently, the sections were rinsed with PBS and incubated with an HRP-conjugated goat anti-rabbit secondary antibody at 37 °C for 20 min. HRP activity was detected using diaminobenzidine (Invitrogen, Carlsbad, CA, USA), and the sections were counterstained with haematoxylin. Control sections were run concurrently with the experimental sections using nonspecific rabbit IgG, and they were similarly pretreated. Nonspecific staining was not detected in the controls.

### Mouse model of adenomyosis

All experiments involving animals were approved by the Institutional Animal Care and Use Committee of Nanjing Drum Tower Hospital. ICR mice were purchased from the experimental animal centre of Yangzhou University. On the second day after birth, ICR mice were separated from their mothers in the early morning. After 6 h of starvation, they were treated with 5 μL/g peanut oil/lecithin/condensed milk mixture (2:0.2:3) supplemented with tamoxifen (1.52 mg/(kg.bw)) for three consecutive days. After drip feeding, they were returned to the female cage. From the 22nd day, they were separated from the female and fed freely. Some mice needed to be sacrificed for the study. The method of sacrifice was cervical dislocation after induced anaesthesia and sedation by pentobarbital sodium. The uteri of 2-month old mice were stained with H&E, α-SMA and E-cadherin to observe the invasion of glands into the muscle layer and disordered of muscle layer proliferation.

### Statistical analysis

The data are presented as the means ± SEM. All experiments were performed at least three times. Student’s t test was used for comparisons between two groups. Statistical analysis was conducted by ANOVA, followed by the Student–Newman–Keuls test, for experiments involving more than two groups. Pearson correlation analysis was used to assess the relationship between KLF4 and Beclin-1; and LC3-B/LC3-A. P values of less than 0.05 were considered statistically significant.

## Results

### Aberrant expression of KLF4, LC3-B/LC3-A, and Beclin-1 in the endometria of patients with adenomyosis

**Fig. 1:**
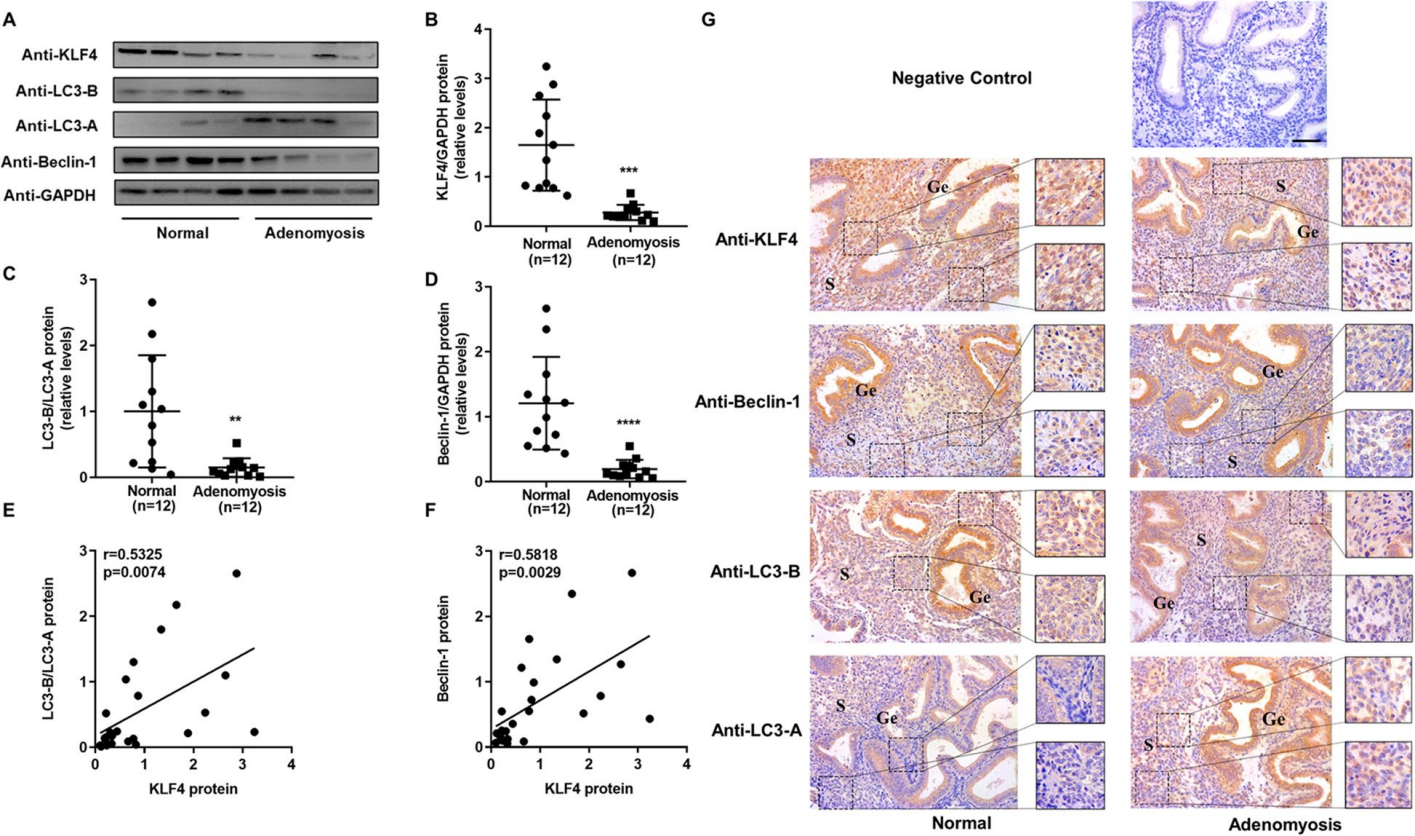
Aberrant expression of KLF4 and autophagy markers in the endometrium of patients with adenomyosis. **A** Differences in protein expression in endometrial samples were assessed by Western blotting using antibodies specific to KLF4, LC3-B, LC3-A and Beclin-1. **B**, **C** and **D** The total KLF4, LC3-B/LC3-A and Beclin-1 protein levels were normalized to GAPDH expression, and the data for all of the endometrial samples are shown in scatter plots. ^**^P < 0.01, ^***^P < 0.001 and.^****^P < 0.0001 compared with the fertile group. **E** Correlation between KLF4 and LC3-B/LC3-A protein expression (*n* = 24, *r* = 0.5325, *P* = 0.0074). **F** Correlation between KLF4 and Beclin-1 protein expression (*n* = 24, *r* = 0.5815, *P* = 0.0029). **G** Immunohistochemical analysis was performed using KLF4, LC3-B, LC3-A and Beclin-1 antibodies. Endometrial tissue samples from fertile women and adenomyosis patients are shown at 400 × magnification, and parts of the images were enlarged 2 times. The negative control was nonspecific rabbit serum. Scale bar, 50 μm

We investigated the expression patterns of related molecules in the endometria of infertile women with adenomyosis and normal fertile women. As shown in Fig. 1, the KLF4 protein level in the endometria of patients with adenomyosis was significantly lower than that in the endometria of normal controls (Fig. 1A and B). Similarly, we also detected reduced protein levels of the autophagy markers LC3-B/LC3-A and Beclin-1 (Fig. 1A, C and D). In addition, we analysed the correlation between KLF4 protein levels and LC3-B/LC3-A or Beclin-1 protein levels and found a moderate positive correlation (Fig. 1E and F). Furthermore, immunohistochemical analysis showed that the expression levels of KLF4, LC3-B and Beclin-1 in endometrial stromal cells of patients with adenomyosis were significantly decreased, while LC3-A levels were not decreased (Fig. 1G). Therefore, the expression of KLF4 and the autophagy markers LC3-B/LC3-A and Beclin-1 was abnormally decreased in the endometria of adenomyosis patients, and there was a moderate positive correlation between KLF4 and autophagy markers (LC3-B/LC3-A and Beclin-1).

### KLF4 induces decidualization in vitro

**Fig. 2:**
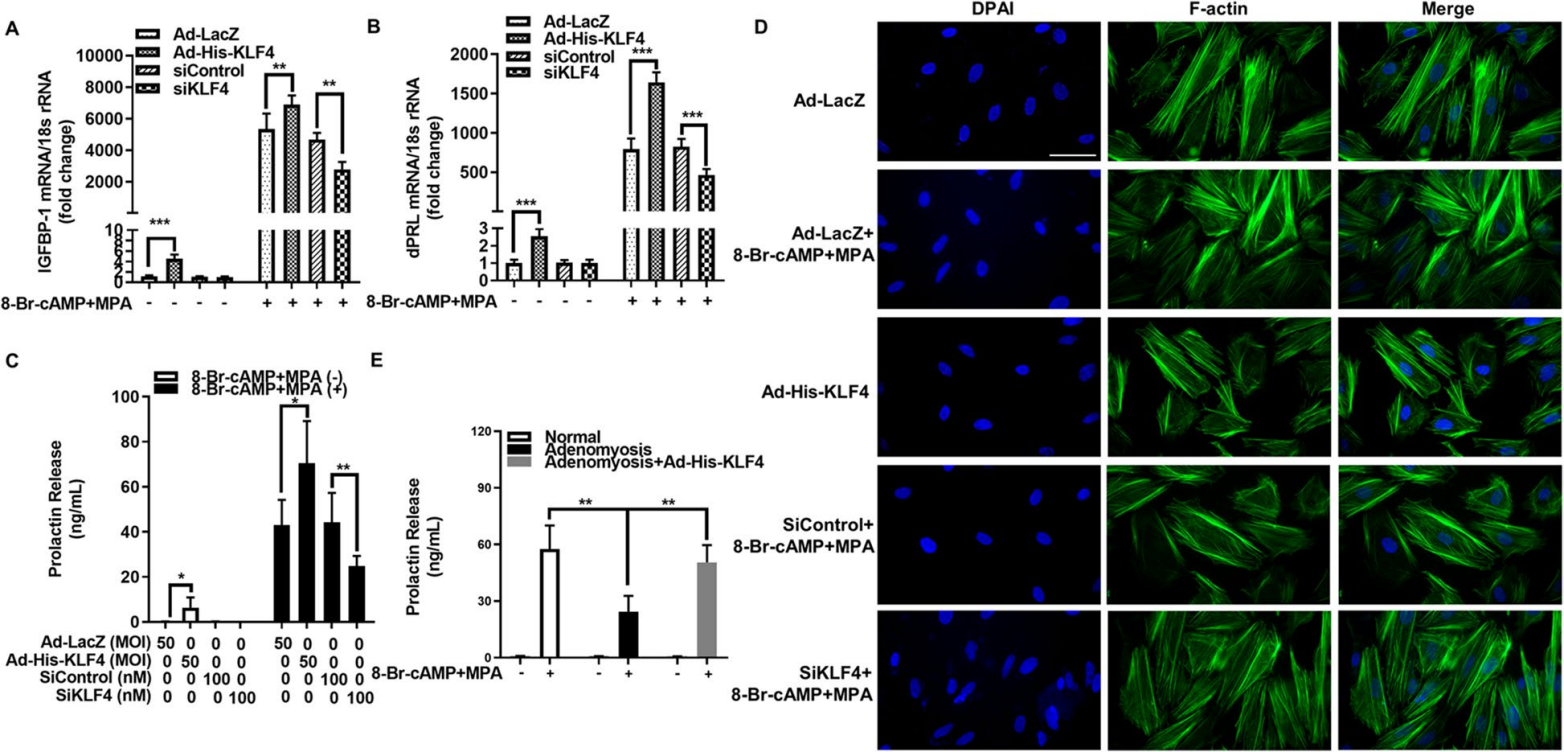
KLF4 enhances 8-Br-cAMP and MPA-induced hESC decidualization in vitro. **A** and **B** hESCs (from fertile controls, *n* = 3) were infected with Ad-His-KLF4 or Ad-LacZ (MOI = 50) and transfected with SiControl or SiKLF4 (100 nM) for 24 h followed by treatment with 0.5 mM 8-Br-cAMP and 1 μM MPA for 3 days. dPRL and IGFBP-1 mRNA levels were measured by qRT–PCR. ^**^P < 0.01, ^***^P < 0.001. **C** hESCs (from fertile controls, *n* = 3) were infected with Ad-His-KLF4 or Ad-LacZ (MOI = 50) and transfected with SiControl or SiKLF4 (100 nM) for 24 h followed by treatment with 0.5 mM 8-Br-cAMP and 1 μM MPA for 3 days. Prolactin release into the medium was measured by an enzyme-linked fluorescent assay (ELFA). ^*^P < 0.05, ^**^P < 0.01. **D** hESCs from fertile controls were infected with Ad-LacZ or Ad-His-KLF4 (MOI = 50) and transfected with SiControl or SiKLF4 (100 nM). After 24 h, the cells were treated with 0.5 mM 8-Br-cAMP and 1 μM MPA as indicated for an additional 72 h. Fluorescein isothiocyanate-labelled phalloidin was used to label actin filaments and to analyse the morphological transformation of hESCs. Scale bar, 50 μm. **E** hESCs (from adenomyosis patients, n = 3) were infected with Ad-His-KLF4 or Ad-LacZ (MOI = 50) for 24 h followed by treatment with 0.5 mM 8-Br-cAMP and 1 μM MPA for 3 days. Prolactin release into the medium was measured by an enzyme-linked fluorescent assay (ELFA). ^**^P < 0.01

To explore whether KLF4 can regulate decidualization, KLF4-overexpressing adenovirus (Ad-His-KLF4) and KLF4-knockdown siRNA (SiKLF4) (Fig. S1) were used to pretreat hESCs. After adding 8-Br-cAMP and MPA, the mRNA levels of decidual prolactin (dPRL) and insulin-like growth factor binding protein 1 (IGFBP-1) were significantly increased in the Ad-His-KLF4 + 8-Br-cAMP and MPA groups compared with the 8-Br-cAMP and MPA alonegroups were obviously decreased in the SiKLF4 + 8-Br-cAMP and MPA groups (Fig. 2A and B). In addition, KLF4 overexpression increased the secretion of dPRL, while decreased KLF4 expression can repress the secretion of dPRL (Fig. 2C). At the same time, F-actin staining showed that high expression of KLF4 promoted the transformation of hESCs from the slender type to the round secretory type (Fig. 2D). Decreased KLF4 expression also inhibited this decidualized transformation (Fig. 2D). Our results suggested that the expression of KLF4 in the endometrium of patients with adenomyosis was decreased (Fig. 1). We also isolated hESCs from adenomyosis patients. Decidualization was induced in hESCs from both adenomyosis patients and fertile women in vitro. In adenomyotic hESCs, overexpression of KLF4 significantly reversed the decreased secretion of dPRL (Fig. 2E).

### KLF4 induces decidualization by promoting autophagy levels

**Fig. 3:**
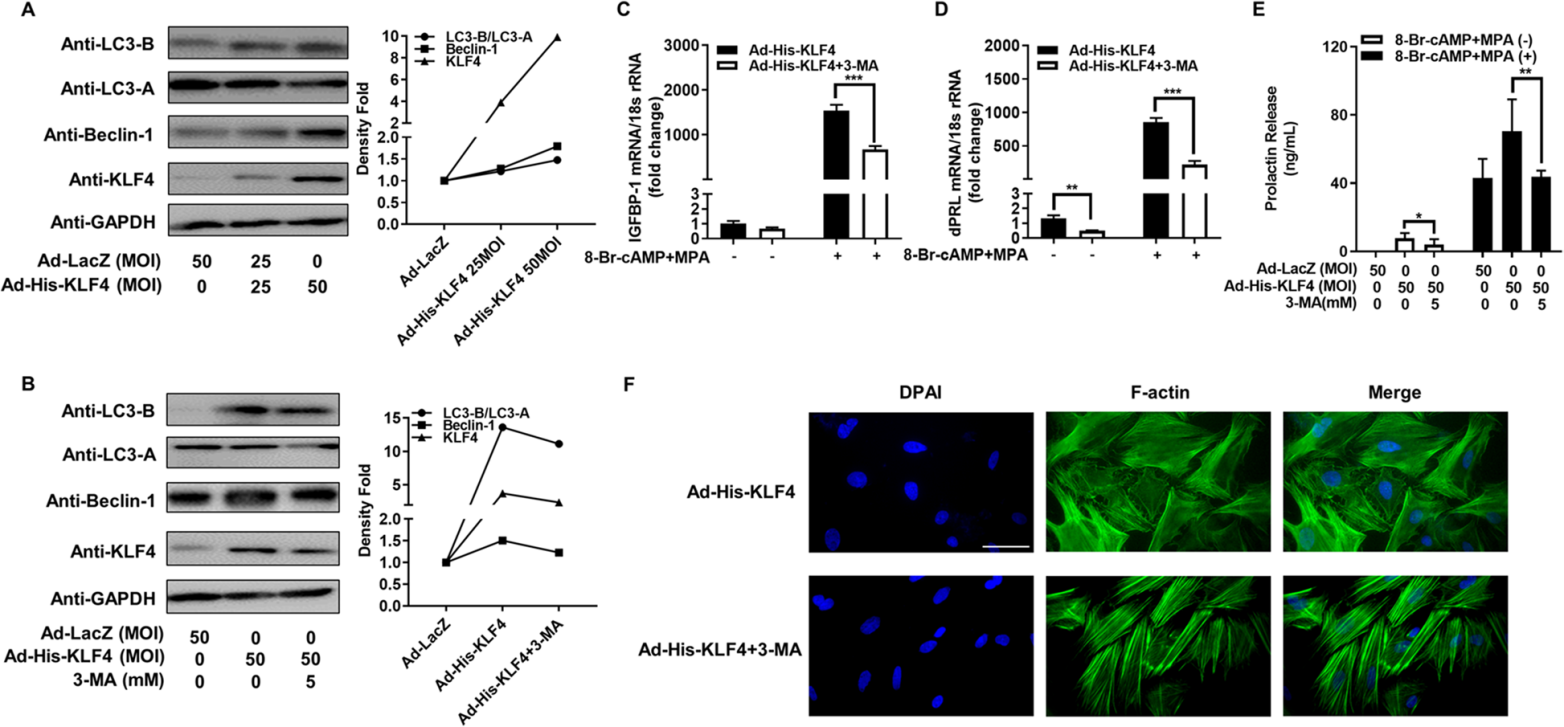
KLF4 enhances 8-Br-cAMP and MPA-induced hESC decidualization by promoting autophagy. **A** HESCs (from fertile controls, *n* = 3) were infected with Ad-His-KLF4 or Ad-LacZ (MOI = 25 and 50) for 48 h. LC3-B, LC3-A, Beclin-1, and KLF4 protein levels were analysed by western blot. **B** hESCs (from fertile controls, *n* = 3) were infected with Ad-His-KLF4 or Ad-LacZ (MOI = 50) for 24 h followed by treatment with 3-MA (5 mM) for 24 h. LC3-B, LC3-A, Beclin-1, and KLF4 protein levels were analysed by western blot. **C** and **D** hESCs (from fertile controls, *n* = 3) were infected with Ad-His-KLF4 (MOI = 50) or Ad-His-KLF4 (MOI = 50) with 3-MA (5 mM) for 24 h followed by treatment with 0.5 mM 8-Br-cAMP and 1 μM MPA for 3 days. dPRL and IGFBP-1 mRNA levels were measured by qRT–PCR. ^**^P < 0.01, ^***^P < 0.001. **E** hESCs (from fertile controls, *n* = 3) were infected with Ad-LacZ (MOI = 50), Ad-His-KLF4 (MOI = 50) or Ad-His-KLF4 (MOI = 50) with 3-MA (5 mM) for 24 h followed by treatment with 0.5 mM 8-Br-cAMP and 1 μM MPA for 3 days. Prolactin release into the medium was measured by an enzyme-linked fluorescent assay (ELFA). ^*^P < 0.05, ^**^P < 0.01. **F** hESCs from fertile controls were infected with Ad-His-KLF4 (MOI = 50) or Ad-His-KLF4 (MOI = 50) with 3-MA (5 mM). After 24 h, the cells were treated with 0.5 mM 8-Br-cAMP and 1 μM MPA as indicated for an additional 72 h. Fluorescein isothiocyanate-labelled phalloidin was used to label actin filaments and to analyse the morphological transformation of hESCs. Scale bar, 50 μm

Overexpression of KLF4 in hESCs could promoted autophagy, as detected by increased expression of LC3-B/LC3-A and Beclin-1 (Fig. 3A). As shown in Fig. 3B, an autophagy inhibitor (3-MA) inhibited the KLF4-induced autophagy marker protein levels (LC3-B/LC3-A and Beclin-1). 3-MA also inhibited the mRNA levels of dPRL and IGFBP-1 induced by KLF4 overexpression (Fig. 3C and D). At the same time, the secretion level of dPRL in hESCs with higher KLF4 expression could be repressed by 3-MA (Fig. 3E). In addition, F-actin cytoskeleton staining suggested that 3-MA treatment could reverse the effect of KLF4 on the decidualization of hESC morphological changes (Fig. 3F). Therefore, KLF4 promotes decidualization of hESCs by regulating their autophagy level.

### KLF4 promotes autophagy levels by transcriptionally increasing ATG5

**Fig. 4:**
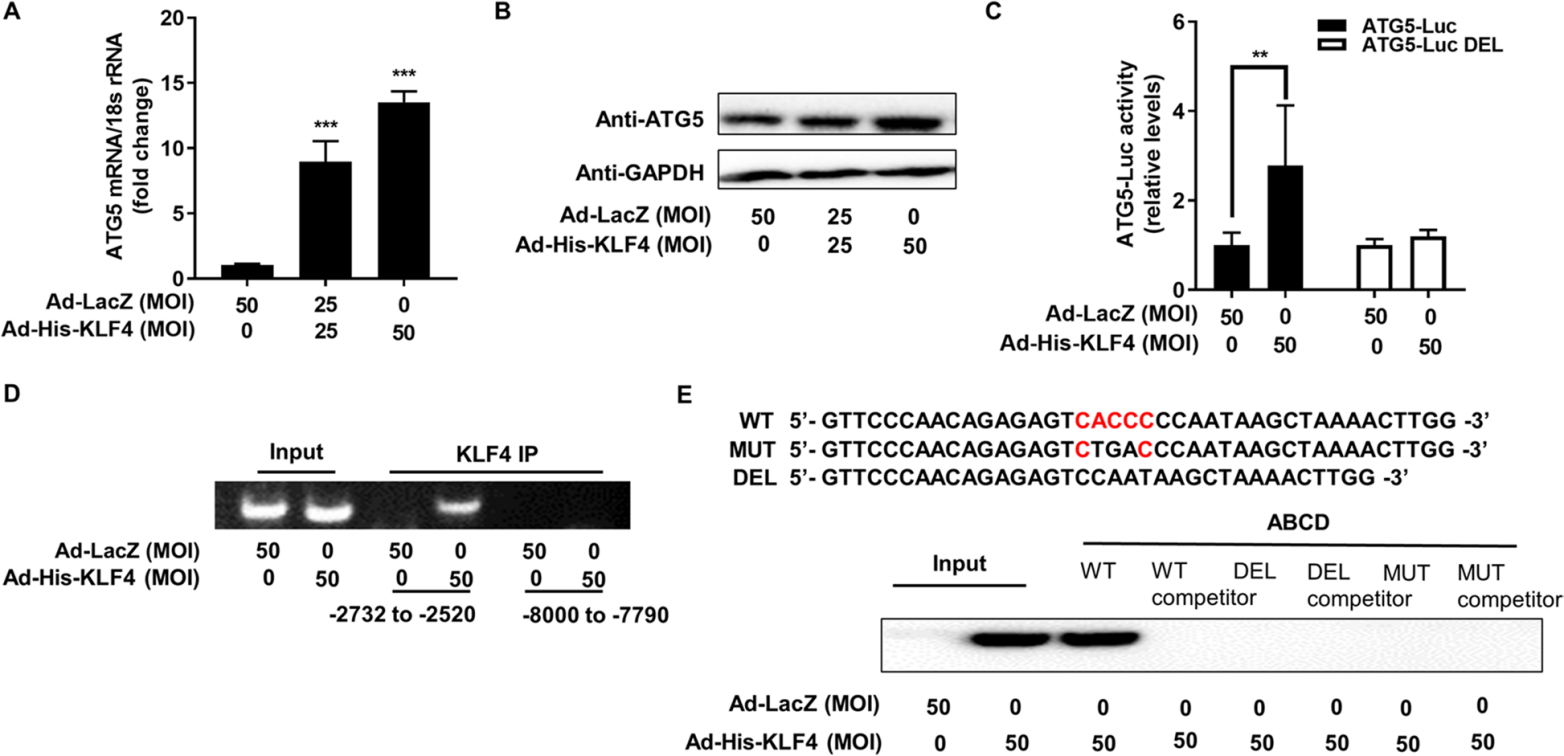
KLF4 directly induces ATG5 transcription. **A** and **B** hESCs (from fertile controls, *n* = 3) were infected with Ad-LacZ or Ad-His-KLF4 (MOI = 25 and 50) for 48 h. ATG5 mRNA and protein levels were measured by qRT–PCR and Western blotting, respectively. ^***^P < 0.001. **C** hESCs (from fertile controls, *n* = 3) were infected with Ad-LacZ or Ad-His-KLF4 (MOI = 50) for 48 h and then transfected with ATG5-Luc or ATG5-Luc deletion (300 ng/well). After 48 h, luciferase assays were performed, and the data were plotted after normalization to Renilla luciferase activity. ^**^P < 0.01. **D** ChIP-PCR amplification using primers against the human ATG5 promoter region. PCR products were separated by agarose gel electrophoresis. Input (nonprecipitated) chromatin was utilized as a positive control in these analyses. **E** ABCD assays were performed using biotinylated or nonbiotinylated (competitor) double-stranded ATG5 wild-type (WT), conserved element-deleted (DEL) and conserved element-mutated (MUT) oligonucleotides with whole-cell extracts from hESCs infected with Ad-His-KLF4 or Ad-LacZ (MOI = 50) for 48 h

To further clarify how KLF4 regulates the autophagy level of hESCs, we found that KLF4 could promote the expression of ATG5 at the mRNA and protein levels (Fig. 4A and B). In addition, we analysed the promoter region of ATG5, a key autophagy regulator, and found that this region contained a specific binding sequence of KLF4. Therefore, we constructed luciferase reporter plasmids containing the specific binding sites (ATG5-Luc) and without the binding sites (ATG5-Luc-DEL). The luciferase reporter assay showed that KLF4 promoted the luciferase activity of ATG5-Luc-WT, but had no significant effect on the deleted plasmid (ATG5-Luc-DEL) (Fig. 4C). Next, we performed ChIP-PCR analysis to investigate whether the ATG5 promoter is a direct binding target of KLF4 in hESCs. As shown in Fig. 4D, the promoter DNA fragment (-2732 to -2520 bp) could be effectively enriched and amplified from the immunoprecipitation complex of the Ad-His-KLF4 protein, which was more than that enriched from the Ad-LacZ control group. In addition, no specific PCR products (-8000 to -7790 bp) were obtained from the Ad-His-KLF4 or Ad-LacZ immunoprecipitated complex using negative control primers. In addition, ABCD assays were performed using biotinylated double-stranded oligonucleotides corresponding to the WT (− 2644/ − 2605 bp), deleted (DEL) and mutated (MUT) ATG5 promoter sequences. The results showed that the KLF4 proteins strongly bound to the WT probe but not to the DEL or MUT probe (Fig. 4E).

### Aberrant expression of KLF4, LC3-B/LC3-A, and Beclin-1 in the uterus of adenomyotic mice

**Fig. 5:**
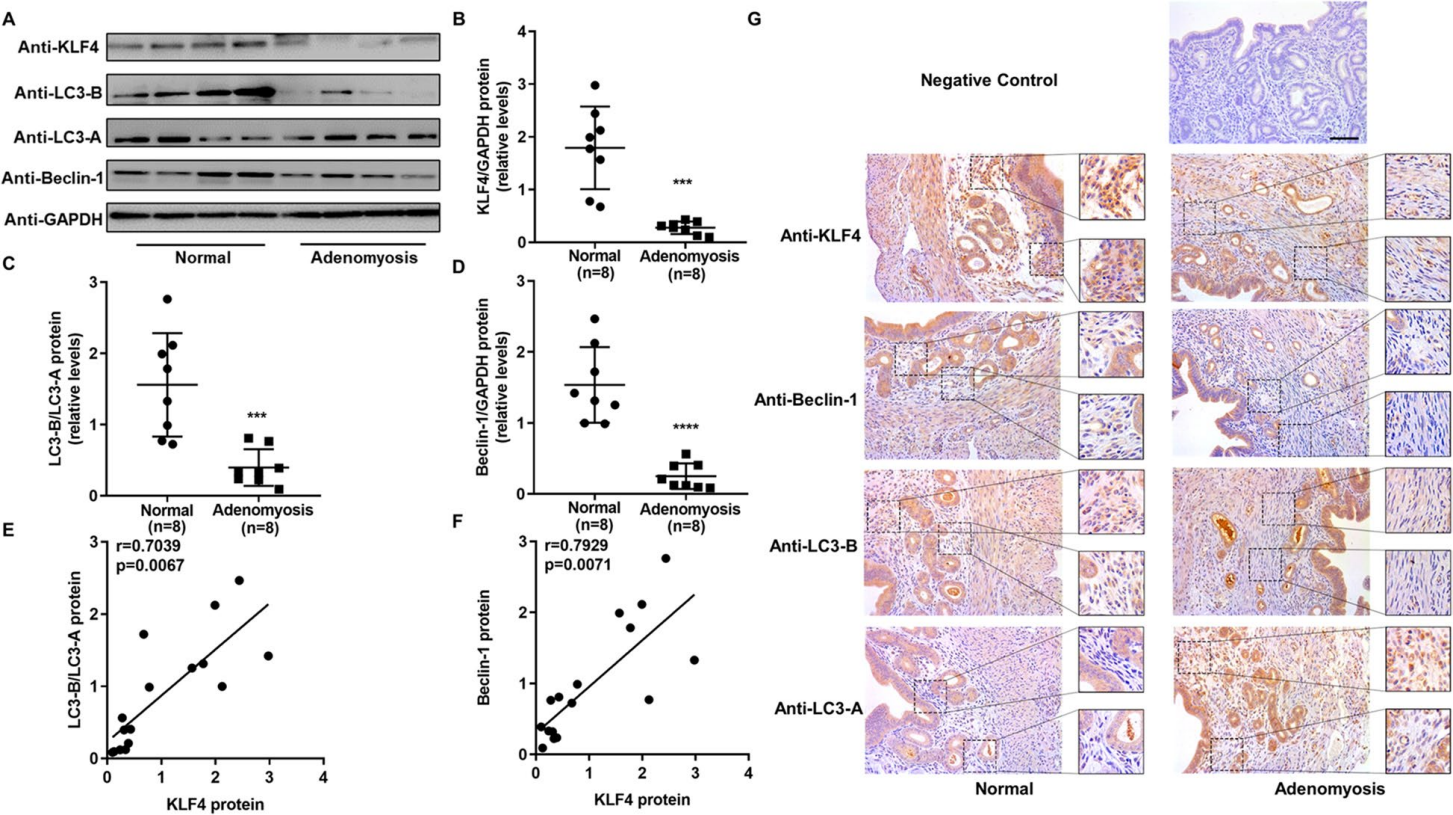
Aberrant expression of KLF4 and autophagy markers in the uteri of adenomyotic mice. **A** Differences in protein expression in uterine samples were assessed by Western blotting using antibodies specific to KLF4, LC3-B, LC3-A and Beclin-1. **B**, **C** and **D** The total KLF4, LC3-B/LC3-A and Beclin-1 protein levels were normalized to GAPDH expression, and the data for all of the uterus samples are shown in the scatter plots. ^***^P < 0.001 and.^****^P < 0.0001 compared with the fertile group. **E** Correlation between KLF4 and LC3-B/LC3-A protein expression (*n* = 16, r = 0.7039, *P* = 0.0067). **F** Correlation between KLF4 and Beclin-1 protein expression (*n* = 16, *r* = 0.7929, *P* = 0.0071). **G** Immunohistochemical analysis was performed using KLF4, LC3-B, LC3-A and Beclin-1 antibodies. Uterus tissue samples from adenomyosis and normal mice are shown at 400 × magnification, and parts of the images were enlarged 2 times. The negative control was nonspecific rabbit serum. Scale bar, 50 μm

To further explore the expression pattern of KLF4 and autophagy markers in vivo, we used tamoxifen to construct an adenomyosis mouse model. By H&E staining and immunofluorescence staining of α-SMA and E-cadherin, we found that the myometrium was disordered, and the endometrial glands invaded in the myometrium, which suggested that the adenomyosis mouse model was successfully constructed (Fig. S2). Similar to the endometrial samples of adenomyosis patients, we detected abnormally decreased expression of KLF4 and autophagy markers (LC3-B/LC3-A and Beclin-1) in the uteri of adenomyosis mice (Fig. 5A-D). In addition, the expression of KLF4 was positively correlated with LC3-B/LC3-A and Beclin-1 (Fig. 5E and F). The immunohistochemical staining showed similar results (Fig. 5G).

## Discussion

Adenomyosis is closely related to the pregnancy outcome of infertile patients who need IVF treatment and mainly manifests as embryo implantation failure or early miscarriage [1–3]. However, the exact mechanism by which adenomyosis affects pregnancy outcomes is still unclear. In recent years, the control of embryo quality in IVF cycles has improved gradually; meanwhile, endometrial dysfunction (such as decreased endometrial receptivity to embryo adhesion or impaired decidualization) is increasingly considered to be one of the main factors limiting successful pregnancy among patients with adenomyosis [10, 29, 30]. Pregnancy is a complex and precise process, that requires perfect cooperation between the embryo and endometrium [31–34]. Decidual endometrial stromal cells secrete matrix metalloproteinase (MMP) family molecules and tissue inhibitors of metalloproteinases (TIMPs), which work together to maintain the moderate invasion process of trophoblasts. MMPs promote the invasion process, while TIMPs prevent the excessive invasion [35, 36]. In addition, decidual stromal cells play another role in information transmission between immune cells [37] and provide the necessary immune tolerance microenvironment for embryo implantation [38]. Decidualization of endometrial stromal cells plays an important role in embryo implantation and pregnancy maintenance. Therefore, impaired decidualization may be an important reason for the lower rate of embryo implantation and higher rate of miscarriage in infertile patients with adenomyosis. In addition, our previous studies found that the expression of the orphan nuclear receptor family member NR4A1 in endometrial stromal cells of adenomyosis patients was significantly decreased, which inhibited the transcriptional activation of FOXO1A, leading to inhibition of the decidualization process [11].

Krüppel-like factor family members are evolutionarily conserved DNA binding proteins and important nuclear transcription factors, that can directly bind to specific conserved motifs in the promoter region of target genes and play a role in downstream transcriptional regulation [39, 40]. Through the study of gene knockout mice, it has been confirmed that a variety of endometrial KLFs participate in the process of embryo implantation. The decidualization process of uterine Klf5 knockout mice was significantly inhibited, resulting in the failure of embryo implantation [16, 17] and the embryo implantation rate was cleraly decreased obviously Klf9 knockout mice [18, 19]. Our previous studies confirmed that KLF12 could inhibit the decidualization process by transcriptional repression of NR4A1 and FOXO1A expression [20, 21]. Another KLF family member, KLF4, could be upregulated by progesterone during the preimplantation period, while KLF4 could affect the proliferation and apoptosis of epithelial cells, suggesting that KLF4 might participate in the regulation of embryo implantation [41, 42]. Furthermore, we found that KLF4 can promote endometrial stromal cell decidualization. The expression of KLF4 in endometrial stromal cells of adenomyosis-infertile patients was significantly decreased, which might be an important reason for the impaired decidualization process.

Previous studies have suggested that the level of autophagy in endometrial cells is closely related to early embryo implantation. Compared with normal pregnancy decidua, early abortion decidua is in a state of lower autophagy [22]. In addition, in a mouse model of artificial decidualization, the inhibition of autophagy by 3-methyladenine resulted in impaired decidualization and abnormal expression of progesterone receptor (PR) and HOXA10 protein [43]. Therefore, endometrial autophagy may be necessary for embryo implantation, which is closely related to the decidualization process in early pregnancy. We further detected abnormally decreased expression of autophagy markers (LC3-B/LC3-A and Beclin-1) in the endometria of adenomyosis patients and their expression levels weremoderately positively correlated with KLF4 expression levels. Furthermore, we investigated the effect of KLF4 on the autophagy status of endometrial stromal cells. The results showed that overexpression of KLF4 could promote the expression of autophagy-related proteins and that the autophagy status of KLF4-reduced endometrial stromal cells was significantly repressed. The addition of an autophagy inhibitor (3-MA) significantly reversed the positive effect of KLF4 overexpression on decidualization of the endometrial stromal cells, indicating that KLF4 promotes decidualization by inducing autophagy.

The initiation of autophagy depends on a double membranous vesicle called a phagocyte, which isolates the material in the cytoplasm for lysosomal degradation. The ubiquitin like binding system promotes the elongation and expansion of the phagocyte membrane after phagocytes initiate complex nucleation by autophagy. One of the key binding systems is composed of autophagy related 16 like 1 (ATG16L1), ATG12 and ATG5 [44]. A recent study suggested that ATG16L1 is involved in the regulation of endometrial decidualization and plays an important role in embryo implantation [45]. In addition, the knockdowns of two important ATG proteins, ATG7 and ATG5, impaired decidualization, confirming a positive role of these proteins and of autophagy for the correct decidualization of human endometrial stromal cells [46]. To further explore how KLF4 regulates the autophagy level of endometrial stromal cells, we visually scanned the promoter sequence of the autophagy related protein ATG5 (-3000 to + 100 bp) and found conserved KLF4-binding sites. In addition, we confirmed that KLF4 can directly bind to the promoter region of ATG5 and transcriptionally activate its expression by ChIP-PCR and ABCD experiments. Moreover, inhibition of ATG5 expression could suppress the autophagy status of endometrial stromal cells with high KLF4 expression, which indicated that KLF4 might promote autophagy by transcriptionally inducing ATG5 expression. However, KLF4 may have transcriptional regulation of other autophagy related proteins. Therefore, ChIP sequencing is worthy of further investigation to identify downstream genes of KLF4, which can help us better describe the molecular network of KLF4 regulating the autophagy status of endometrial stromal cells.

We have identified that KLF4 maintains normal autophagy and decidualization of endometrial stromal cells through transcriptional regulation of ATG5 expression. The abnormally decreased expression of KLF4 in endometrial stromal cells of patients with adenomyosis breaks the original balance, which puts endometrial stromal cells in an abnormally low autophagy state and damages the decidualization process, thus impairing embryo implantation and further pregnancy maintenance. However, the immunohistochemical results of the endometrium in adenomyosis patients showed that KLF4 and autophagy-related marker expression were reduced in both endometrial stromal cells, but also in endometrial epithelial cells. Embryo implantation failure may also occur at the stage of embryo adhesion. The abnormal expression of KLF4 in endometrial epithelial cells and its abnormal autophagy condition may also reduce the ability of the endometrium to accept embryo adhesion in adenomyosis patients. Therefore, the specific effect of abnormal KLF4 expression in endometrial epithelial cells and its exact regulatory mechanism need to be further studied. On the other hand, our study only used in vitro experiments to explore the regulatory mechanism and an adenomyosis mouse model for preliminary in vivo investigation. Furthermore, it is necessary to construct a conditional Klf4 knockout mouse model of endometrial epithelial cells and stromal cells. This could be used to fully evaluate the physiological function of KLF4 and the important function of endometrial autophagy in embryo implantation.

## Conclusion

In conclusion, our results highlight the novel role of KLF4 and autophagy in the decidualization of endometrial stromal cells. The expression level of KLF4 in endometrial stromal cells of patients with adenomyosis was abnormally decreased and it was in an abnormally low autophagy state. Our findings may provide a theoretical basis for the mechanism of embryo implantation failure caused by adenomyosis. They may also provide potential biomarkers and therapeutic targets for the decidualization dysfunction associated with adenomyosis.

## Supplementary Information

**Additional file 1: Figure S1.** SiKLF4 represses KLF4 expression in hESCs. A and B hESCs (from fertile controls, *n* = 3) were transfected with SiControl or SiKLF4 (50, 100 nM) for 48 h. KLF4 mRNA and protein levels were measured by qRT-PCR and Western blotting, respectively. ^***^P < 0.001.

**Additional file 2: Figure S2.** Identification of adenomyotic mice. The uterus of 2-month mouse was stained with H&E, α-SMA and E-cadherin to observe the invasion of glands into muscle layer and the disorder of muscle layer proliferation. Scale bar, 50 μm.

## Data Availability

The datasets used and/or analyzed during the current study are available from the corresponding author on reasonable request.

## Abbreviations

KLF4: Krüppel-like factor 4
hESCs: Human endometrial stromal cells
qRT-PCR: Quantitative-real-time PCR
ChIP: Chromatin immunoprecipitation
ABCD: Avidin-biotin conjugate DNA precipitation
ATG5: Autophagy related 5
HOXA10: Homeobox A10
LIF: Leukemia inhibitory factor
mTOR: Mammalian target of rapamycin
IVF: In vitro fertilization
PCOS: Polycystic ovary syndrome
MPA: Medroxyprogesterone acetate
dPRL: Decidual prolactin
3-MA: 3-Methyladenine
PVDF: Polyvinylidene fluoride
IGFBP-1: Insulin-like growth factor binding protein 1
MMP: Matrix metalloproteinase
ATG16L1: Autophagy related 16 like 1

## References

[1] Vercellini P, Consonni D, Dridi D, Bracco B, Frattaruolo MP, Somigliana E (2014). Uterine adenomyosis and in vitro fertilization outcome: a systematic review and meta-analysis. Hum Reprod.

[2] Salim R, Riris S, Saab W, Abramov B, Khadum I, Serhal P (2012). Adenomyosis reduces pregnancy rates in infertile women undergoing IVF. Reprod Biomed Online.

[3] Tremellen K, Russell P (2011). Adenomyosis is a potential cause of recurrent implantation failure during IVF treatment. Aust N Z J Obstet Gynaecol.

[4] Munro MG (2019). Uterine polyps, adenomyosis, leiomyomas, and endometrial receptivity. Fertil Steril.

[5] Cha J, Sun X, Dey SK (2012). Mechanisms of implantation: strategies for successful pregnancy. Nat Med.

[6] Lim HJ, Wang H (2010). Uterine disorders and pregnancy complications: insights from mouse models. J Clin Invest.

[7] Zhang S, Lin H, Kong S, Wang S, Wang H, Wang H, Armant DR (2013). Physiological and molecular determinants of embryo implantation. Mol Aspects Med.

[8] Diedrich K, Fauser BC, Devroey P, Griesinger G, Evian Annual Reproduction Workshop G (2007). The role of the endometrium and embryo in human implantation. Hum Reprod Update.

[9] Simon C, Martin JC, Pellicer A (2000). Paracrine regulators of implantation. Baillieres Best Pract Res Clin Obstet Gynaecol.

[10] Xiao Y, Sun X, Yang X, Zhang J, Xue Q, Cai B, Zhou Y (2010). Leukemia inhibitory factor is dysregulated in the endometrium and uterine flushing fluid of patients with adenomyosis during implantation window. Fertil Steril.

[11] Jiang Y, Jiang R, Cheng X, Zhang Q, Hu Y, Zhang H, Cao Y, Zhang M, Wang J, Ding L, Diao Z, Sun H (2016). Decreased expression of NR4A nuclear receptors in adenomyosis impairs endometrial decidualization. Mol Hum Reprod.

[12] Kajihara T, Tanaka K, Oguro T, Tochigi H, Prechapanich J, Uchino S, Itakura A, Sucurovic S, Murakami K, Brosens JJ, Ishihara O (2014). Androgens modulate the morphological characteristics of human endometrial stromal cells decidualized in vitro. Reprod Sci.

[13] Iwahashi M, Muragaki Y, Ooshima A, Yamoto M, Nakano R (1996). Alterations in distribution and composition of the extracellular matrix during decidualization of the human endometrium. J Reprod Fertil.

[14] Gellersen B, Reimann K, Samalecos A, Aupers S, Bamberger AM (2010). Invasiveness of human endometrial stromal cells is promoted by decidualization and by trophoblast-derived signals. Hum Reprod.

[15] Weimar CH, Macklon NS, Post Uiterweer ED, Brosens JJ, Gellersen B (2013). The motile and invasive capacity of human endometrial stromal cells: implications for normal and impaired reproductive function. Hum Reprod Update.

[16] Sun X, Zhang L, Xie H, Wan H, Magella B, Whitsett JA, Dey SK (2012). Kruppel-like factor 5 (KLF5) is critical for conferring uterine receptivity to implantation. Proc Natl Acad Sci U S A.

[17] Lin SC, Wani MA, Whitsett JA, Wells JM (2010). Klf5 regulates lineage formation in the pre-implantation mouse embryo. Development.

[18] Zhang XL, Zhang D, Michel FJ, Blum JL, Simmen FA, Simmen RC (2003). Selective interactions of Kruppel-like factor 9/basic transcription element-binding protein with progesterone receptor isoforms A and B determine transcriptional activity of progesterone-responsive genes in endometrial epithelial cells. J Biol Chem.

[19] Heard ME, Pabona JM, Clayberger C, Krensky AM, Simmen FA, Simmen RC (2012). The reproductive phenotype of mice null for transcription factor Kruppel-like factor 13 suggests compensatory function of family member Kruppel-like factor 9 in the peri-implantation uterus. Biol Reprod.

[20] Huang C, Jiang Y, Zhou J, Yan Q, Jiang R, Cheng X, Xing J, Ding L, Sun J, Yan G, Sun H (2017). Increased Kruppel-like factor 12 in recurrent implantation failure impairs endometrial decidualization by repressing Nur77 expression. Reprod Biol Endocrinol.

[21] Zhang H, Zhu X, Chen J, Jiang Y, Zhang Q, Kong C, Xing J, Ding L, Diao Z, Zhen X, Sun H, Yan G (2015). Kruppel-like factor 12 is a novel negative regulator of forkhead box O1 expression: a potential role in impaired decidualization. Reprod Biol Endocrinol.

[22] Avagliano L, Terraneo L, Virgili E, Martinelli C, Doi P, Samaja M, Bulfamante GP, Marconi AM (2015). Autophagy in Normal and Abnormal Early Human Pregnancies. Reprod Sci.

[23] Rhee JS, Saben JL, Mayer AL, Schulte MB, Asghar Z, Stephens C, Chi MM, Moley KH (2016). Diet-induced obesity impairs endometrial stromal cell decidualization: a potential role for impaired autophagy. Hum Reprod.

[24] Mei J, Zhu XY, Jin LP, Duan ZL, Li DJ, Li MQ (2015). Estrogen promotes the survival of human secretory phase endometrial stromal cells via CXCL12/CXCR4 up-regulation-mediated autophagy inhibition. Hum Reprod.

[25] Zhang L, Liu Y, Xu Y, Wu H, Wei Z, Cao Y (2015). The expression of the autophagy gene beclin-1 mRNA and protein in ectopic and eutopic endometrium of patients with endometriosis. Int J Fertil Steril.

[26] Choi J, Jo M, Lee E, Kim HJ, Choi D (2014). Differential induction of autophagy by mTOR is associated with abnormal apoptosis in ovarian endometriotic cysts. Mol Hum Reprod.

[27] Yan Q, Yan G, Zhang C, Wang Z, Huang C, Wang J, Zhou J, Liu Y, Ding L, Zhang Q, Zhen X, Jiang Y (2019). miR-21 reverses impaired decidualization through modulation of KLF12 and NR4A1 expression in human endometrial stromal cellsdagger. Biol Reprod.

[28] Huang C, Sun H, Wang Z, Liu Y, Cheng X, Liu J, Jiang R, Zhang X, Zhen X, Zhou J, Chen L, Ding L (2018). Increased Kruppel-like factor 12 impairs embryo attachment via downregulation of leukemia inhibitory factor in women with recurrent implantation failure. Cell Death Discov.

[29] Yen CF, Liao SK, Huang SJ, Tabak S, Arcuri F, Lee CL, Arici A, Petraglia F, Wang HS, Kayisli UA (2017). Decreased Endometrial Expression of Leukemia Inhibitory Factor Receptor Disrupts the STAT3 Signaling in Adenomyosis During the Implantation Window. Reprod Sci.

[30] Xiao Y, Li T, Xia E, Yang X, Sun X, Zhou Y (2013). Expression of integrin beta3 and osteopontin in the eutopic endometrium of adenomyosis during the implantation window. Eur J Obstet Gynecol Reprod Biol.

[31] Wang H, Dey SK (2006). Roadmap to embryo implantation: clues from mouse models. Nat Rev Genet.

[32] Carson DD, Bagchi I, Dey SK, Enders AC, Fazleabas AT, Lessey BA, Yoshinaga K (2000). Embryo implantation. Dev Biol.

[33] Dey SK, Lim H, Das SK, Reese J, Paria BC, Daikoku T, Wang H (2004). Molecular cues to implantation. Endocr Rev.

[34] Macklon NS, Brosens JJ (2014). The human endometrium as a sensor of embryo quality. Biol Reprod.

[35] Martelli M, Campana A, Bischof P (1993). Secretion of matrix metalloproteinases by human endometrial cells in vitro. J Reprod Fertil.

[36] Lockwood CJ, Krikun G, Hausknecht VA, Papp C, Schatz F (1998). Matrix metalloproteinase and matrix metalloproteinase inhibitor expression in endometrial stromal cells during progestin-initiated decidualization and menstruation-related progestin withdrawal. Endocrinology.

[37] Dominguez F, Yanez-Mo M, Sanchez-Madrid F, Simon C (2005). Embryonic implantation and leukocyte transendothelial migration: different processes with similar players?. FASEB J.

[38] van Mourik MS, Macklon NS, Heijnen CJ (2009). Embryonic implantation: cytokines, adhesion molecules, and immune cells in establishing an implantation environment. J Leukoc Biol.

[39] Schuierer M, Hilger-Eversheim K, Dobner T, Bosserhoff AK, Moser M, Turner J, Crossley M, Buettner R (2001). Induction of AP-2alpha expression by adenoviral infection involves inactivation of the AP-2rep transcriptional corepressor CtBP1. J Biol Chem.

[40] Lomberk G, Urrutia R (2005). The family feud: turning off Sp1 by Sp1-like KLF proteins. Biochem J.

[41] Shimizu Y, Takeuchi T, Mita S, Notsu T, Mizuguchi K, Kyo S (2010). Kruppel-like factor 4 mediates anti-proliferative effects of progesterone with G(0)/G(1) arrest in human endometrial epithelial cells. J Endocrinol Invest.

[42] Sahin C, Dilsiz OY, Demiray SB, Yeniel O, Ergenoglu M, Sezer ED, Oktem G, Goker EN, Tavmergen E (2014). Increased stem cell marker expressions during the peri-implantation period in the rat endometrium: constructive role of exogenous zinc and/or progesterone. Biomed Res Int.

[43] Su Y, Zhang JJ, He JL, Liu XQ, Chen XM, Ding YB, Tong C, Peng C, Geng YQ, Wang YX, Gao RF (2020). Endometrial autophagy is essential for embryo implantation during early pregnancy. J Mol Med (Berl).

[44] Romanov J, Walczak M, Ibiricu I, Schuchner S, Ogris E, Kraft C, Martens S (2012). Mechanism and functions of membrane binding by the Atg5-Atg12/Atg16 complex during autophagosome formation. EMBO J.

[45] Oestreich AK, Chadchan SB, Popli P, Medvedeva A, Rowen MN, Stephens CS, Xu R, Lydon JP, Demayo FJ, Jungheim ES, Moley KH, Kommagani R. The Autophagy Gene Atg16L1 is Necessary for Endometrial Decidualization. Endocrinology 2020; 161. 10.1210/endocr/bqz039 PMC6986551 31875883

[46] Mestre Citrinovitz AC, Strowitzki T, Germeyer A. Decreased Autophagy Impairs Decidualization of Human Endometrial Stromal Cells: A Role for ATG Proteins in Endometrial Physiology. Int J Mol Sci 2019; 20. 10.3390/ijms20123066 PMC6628477 31234569

