# Supplementary figures and images for: Decreased Krüppel-like factor 4 in adenomyosis impairs decidualization by repressing autophagy in human endometrial stromal cells
Source: BMC Mol Cell Biol. 2022 Jun 27;23:24. doi: 10.1186/s12860-022-00425-6 (PMC9238063; doi:10.1186/s12860-022-00425-6)

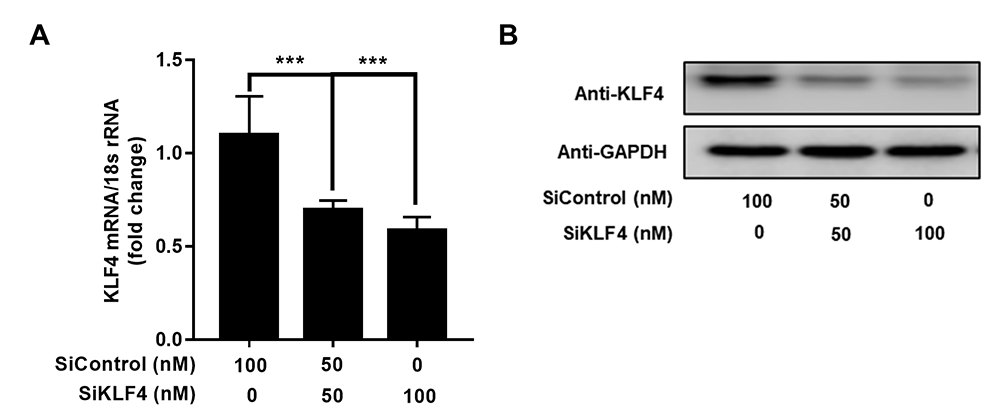

Supplement: Supplementary file 1 — Additional file 1: Figure S1. SiKLF4 represses KLF4 expression in hESCs. A and B hESCs (from fertile controls, n = 3) were transfected with SiControl or SiKLF4 (50, 100 nM) for 48 h. KLF4 mRNA and protein levels were measured by qRT-PCR and Western blotting, respectively. ***P < 0.001. [file 12860_2022_425_MOESM1_ESM.tif]

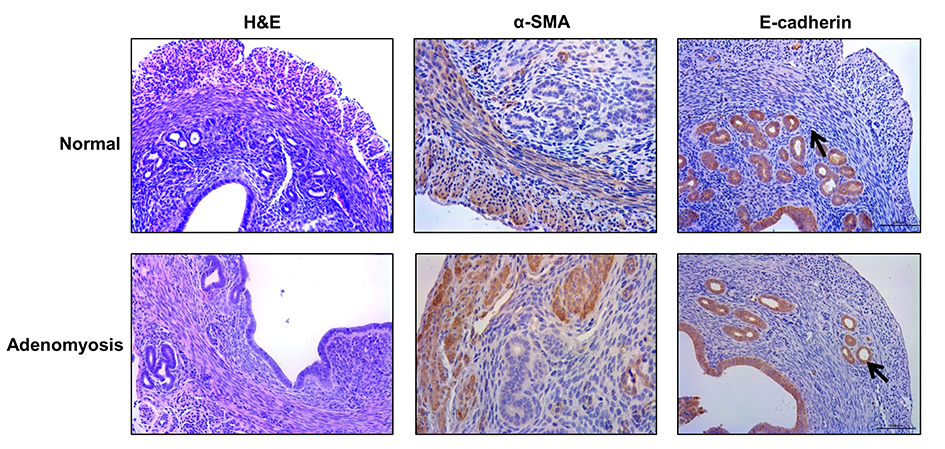

Supplement: Supplementary file 2 — Additional file 2: Figure S2. Identification of adenomyotic mice. The uterus of 2-month mouse was stained with H&E, α-SMA and E-cadherin to observe the invasion of glands into muscle layer and the disorder of muscle layer proliferation. Scale bar, 50 μm. [file 12860_2022_425_MOESM2_ESM.tif]
